# Effects of Non-Natural Amino Acid Incorporation into the Enzyme Core Region on Enzyme Structure and Function

**DOI:** 10.3390/ijms160922735

**Published:** 2015-09-21

**Authors:** H. Edward Wong, Inchan Kwon

**Affiliations:** 1 Department of Chemical Engineering, University of Virginia, Charlottesville, VA 22904, USA; E-Mail: ewong2004@gmail.com; 2 School of Materials Science and Engineering, Gwangju Institute of Science and Technology (GIST), Gwangju 61005, Korea

**Keywords:** non-natural amino acids, enzyme engineering, enzyme kinetics, site-specific incorporation

## Abstract

Techniques to incorporate non-natural amino acids (NNAAs) have enabled biosynthesis of proteins containing new building blocks with unique structures, chemistry, and reactivity that are not found in natural amino acids. It is crucial to understand how incorporation of NNAAs affects protein function because NNAA incorporation may perturb critical function of a target protein. This study investigates how the site-specific incorporation of NNAAs affects catalytic properties of an enzyme. A NNAA with a hydrophobic and bulky sidechain, 3-(2-naphthyl)-alanine (2Nal), was site-specifically incorporated at six different positions in the hydrophobic core of a model enzyme, murine dihydrofolate reductase (mDHFR). The mDHFR variants with a greater change in van der Waals volume upon 2Nal incorporation exhibited a greater reduction in the catalytic efficiency. Similarly, the steric incompatibility calculated using RosettaDesign, a protein stability calculation program, correlated with the changes in the catalytic efficiency.

## 1. Introduction

Protein engineering frequently creates useful proteins with improved or new functions. Successful protein engineering is often governed by the size of amino acid sequence space available for engineering. The advent of technology to genetically encode and incorporate non-natural amino acids (NNAAs) into proteins greatly expanded the amino acid sequence space by increasing the number of residues available for protein biosynthesis. Currently, over 100 NNAAs have been successfully incorporated into various protein targets [1,2,3,4,5]. A significant advantage of using NNAAs is that they possess unique physical, chemical, and biological properties that natural amino acids do not have.

Site-specific incorporation of NNAAs enables the introduction of NNAAs into a specific site of a target protein for a range of protein engineering applications. For example, fluorinated NNAAs have been used as NMR probes to determine the structure and function of nitroreductase and histidinol dehydrogenase [6]. Tyrosine, phenylalanine and tryptophan analogues, (*p*-amino-l-phenylalanine, *p*-methoxy-l-phenylalanine, *p*-iodo-l-phenylalanine, *p*-bromo-l-phenylalanine, and 3-(2-naphthyl)-alanine) have been used to substitute tyrosine at position 66 inside the chromophore of a green fluorescent protein (GFP) [7,8,9,10]. These substitution mutations altered the spectral properties of GFP, yielding shifts in fluorescence emission wavelengths corresponding to the strength of each NNAA as an electron donor [7,8,9,10]. Recently, the use of NNAAs has also been applied to improving the properties of enzymes beyond the limits of natural amino acids. Site-specific incorporation of *p*-aminophenylalanine at Phe124 of nitroreductase (NTR) increased the activity of NTR two-fold over the best natural amino acid mutation [11]. Further, a group of bioorthogonally reactive NNAAs, containing terminal ketones, azides, and alkynes have been developed for bioconjugation applications [12,13,14,15,16,17,18,19]. Since NNAA incorporation can be targeted to specific locations in a protein, NNAAs can serve as a chemical handle generating homogeneous conjugation products, which are often very challenging using only natural amino acids, such as cysteine and lysine [20,21,22,23]. Bioconjugation of fatty acids and polyethylene glycol at reactive NNAA sites improved the blood-circulation longevity of pharmaceutically relevant enzymes and antibodies without significant loss of function [24,25,26].

NNAAs have also been used to alter enzyme properties. In CYP102A1, a catalytically promiscuous cytochrome P450, four aromatic NNAAs were site-specifically incorporated at 11 active-site positions, which resulted in certain mutants exhibiting significant shifts in regioselectivity and improvements to catalytic activity [27]. In addition, the replacement of phenylalanine (Phe) at position 31 of murine dihydrofolate reductase with 3-(2-naphthyl)-alanine or *p*-bromo-phenylalanine led to the alteration of the specificity of murine dihydrofolate reductase towards a less favorable substrate, folate, and reduction of the binding affinity to an inhibitor (methotrexate) [1,28]. In general, one of the big challenges in enzyme engineering is to accurately predict how mutations will affect enzyme function. This is even more challenging when non-natural amino acids are used to engineer enzymes. Therefore, this study investigates how site-specific incorporation of NNAAs into enzymes affects structure and function of the enzymes. Enzymes are a particularly attractive test platform, since enzyme function can be considered a two-step process involving an obligate substrate-binding step and a catalysis step. Both of these steps can be characterized to evaluate the effects of NNAA incorporation on function. In this study, murine dihydrofolate reductase (mDHFR) was used as a model enzyme. There are numerous crystal structures for DHFR, and the enzyme kinetics was well characterized [29,30,31,32,33,34,35,36,37,38,39,40]. The function of mDHFR was characterized though the saturation kinetics of dihydrofolate (DHF) conversion to tetrahydrofolate (THF) which was fit to the Michaelis-Menten equation to obtain the kinetic parameters *K*_m_ and *k*_cat_, to evaluate the effect of NNAA incorporation on the binding and biocatalytic components of mDHFR function, respectively. The study focused primarily on the effect of site-specific incorporation of NNAAs into the hydrophobic core region, where a mutation can significantly perturb the local and/or global structure of the enzyme. In order to minimize direct interactions of a mutated residue with any substrate, active site residues were excluded from mutation.

Since enzymes require their correctly folded structure to function properly, we hypothesized that mutations minimizing structural changes would have a lesser perturbation on enzyme function [41,42,43,44,45,46,47,48,49,50,51,52,53,54]. Since the structure of the hydrophobic core can be governed by its conformational stability, six NNAA incorporation sites were selected from the hydrophobic core of the mDHFR in order to investigate how incorporation of a hydrophobic non-natural amino acid affects the conformational stability and catalytic function of mDHFR. In order to investigate the effect of mutation within the core, we looked at the available hydrophobic NNAAs that had previously been incorporated successfully [28,55,56,57]. Of those, two hydrophobic NNAAs, *p*-bromophenylalanine (pBrF) and 3-(2-naphthyl)-alanine (2Nal) and their corresponding engineered aminoacyl tRNA synthetase/tRNA pairs were available for this work [10,55,58]. While the lower size spectrum of hydrophobic residues is well covered by the natural amino acids in nature, the use of larger sidechains is limited. This presented an opportunity to investigate whether larger hydrophobic amino acids could be successfully incorporated into the enzyme core region, while maintaining enzyme function. 2Nal, a bulky hydrophobic Phe/Trp analogue, was selected for this purpose; it has two conjugated rings and is larger in mass and van der Waals volume than any natural amino acid, as well as pBrF (Supplementary Information Figure S1) [59]. Since 2Nal is bulkier than any natural amino acids, incorporation of 2Nal into the hydrophobic core of mDHFR was expected to significantly perturb the structure and stability of the mDHFR.

## 2. Results and Discussion

### 2.1. Selection of Hydrophobic-Core Incorporation Sites

To select mutation sites, we calculated the accessible solvent area (ASA), a common practice used to locate residues of the hydrophobic core [44,60]. Since solvent accessibility is highly restricted within this area, we restricted our selection to residues (PDB ID: 2W3M) with an ASA less than or equal to 2.0% [44,60]. To eliminate bias that can be associated with using only a single algorithm, we calculated ASA values using three different published calculators to ensure that values were in close accordance with one another (ASA-View, CUPSAT and SDM) [61,62,63]. The ASA cutoff criteria limited selection to five hydrophobic regions. While these selection criteria may not encompass all core residues, this method nearly guarantees the selection of only hydrophobic core residues [44,60,64]. Moreover, we excluded active site and binding pocket residues in order to minimize direct interaction with any substrate or cofactor. With the aforementioned criteria, selection was narrowed to the β-strand portion of the enzyme core. We then carefully selected three pairs of adjacent mutations sites, one pair from each region, for site-specific incorporation of 2Nal. Between variants of adjacent sites, confounding variables—such as differences in location-dependent behavior and local structural environment—were expected to be minimized, which would facilitate pairwise comparison [41,42,44,65,66]. Each adjacent pair of mutation sites consisted of a small valine residue and larger hydrophobic residue. Valine was selected as the smaller residue since it represents an intermediate hydrophobic residue size. The size difference between valine and the adjacent residue allowed a comparison to determine whether the residue size difference between the mutant and native residue (SDMNR) was an important variable that affected enzyme function. Positions Val50, Ile51, Val112, Trp113, Phe134, and Val135 were selected (based on position numbering for PDB:ID 2W3M). Therefore, six mDHFR variants were created, by site-specifically incorporating 2Nal only at the target site. Since 2Nal is an analogue to Trp and Phe, Trp113 and Phe134 serve as conservative mutation sites. Hereinafter mutations are denoted as, for example, V135Z, where natural amino acids are denoted as single letters, and Z indicates 2Nal.

### 2.2. Expression and Purification of mDHFR Variants

The six mDHFR variants (mDHFR^V50Z^, mDHFR^I51Z^, mDHFR^V112Z^, mDHFR^W113Z^, mDHFR^F134Z^, and mDHFR^V135Z^) as well as mDHFR^WT^ were expressed and purified as described previously; though, mutation sites were different [1]. The mDHFR bands at 23.5 kDa were clearly shown in the protein gel (Supplementary Information Figure S2). In the basis of culture volume, the yields of purified mDHFR^WT^, mDHFR^W113Z^, and mDHFR^F134Z^ were 4.7, 2.1, and 1.3 mg/L, respectively. The yields of purified mDHFR^V50Z^, mDHFR^I51Z^, mDHFR^V112Z^, and mDHFR^V135Z^ were 0.7, 0.8, 1.1, and 1.0 mg/L, respectively, which are smaller than those of the other three variants (mDHFR^WT^, mDHFR^W113Z^, and mDHFR^F134Z^). We speculate that the variants with a lower yield (mDHFR^V50Z^, mDHFR^I51Z^, mDHFR^V112Z^, and mDHFR^V135Z^) are not structurally stable inside cells and so prone to cellular degradation leading to relatively poor expression/purification yields.

### 2.3. Verification of in Vivo 2Nal Incorporation by MALDI-TOF/MS

**Figure 1:**
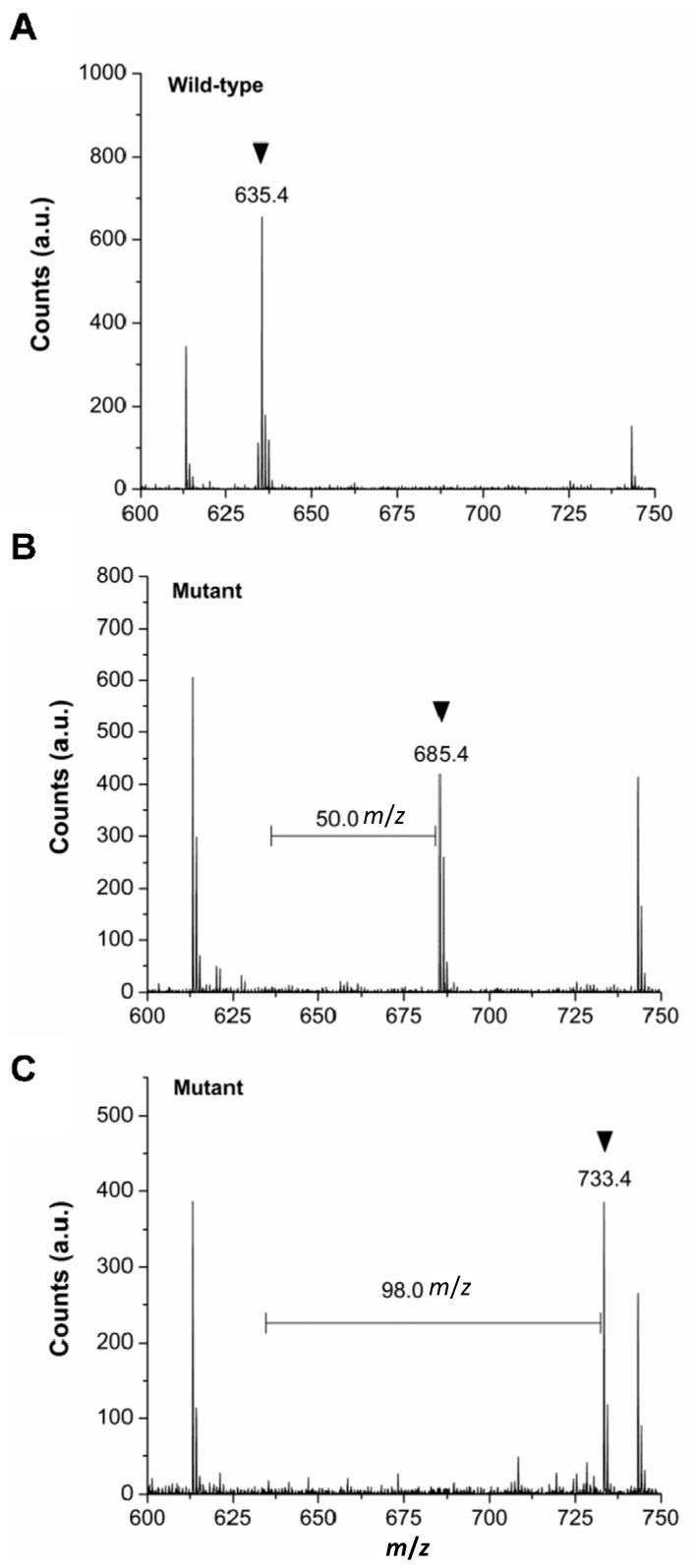
Confirmation of *in vivo* 2Nal incorporation by MALDI-TOF/MS analysis. The MS spectra of a tryptic digest (residues 133–147) for (**A**) mDHFR^WT^; (**B**) mDHFR^F134Z^; and (**C**) mDHFR^V135Z^. The peak corresponding to the 2Nal-containing digest is indicated by the arrow (**B**,**C**). The counterpart mDHFR^WT^ fragment with the native residue is marked by an arrow (**A**). The residue numbering corresponds to PDB ID: 2W3M. The horizontal bar indicates the mass difference between the 2Nal-containing fragment and corresponding mDHFR^WT^ fragment with the native residue. a.u. denotes arbitrary units.

Each variant was digested by either trypsin or chymotrypsin to produce a fingerprint of peptide fragments of known length and mass. Then, *in vivo* site-specific incorporation of 2Nal at each target site was verified by matrix assisted laser desorption ionization-time of flight/mass spectrometry (MALDI-TOF/MS) analysis of the digests. Successful incorporation was expected to yield a peptide fragment containing 2Nal, with monoisotopic mass equivalent to the monoisotopic mass of the native fragment plus the difference in mass between 2Nal and the original residue [1]. As representative cases, site-specific incorporation of 2Nal into F134 and V135 sites were verified by MALDI-TOF/MS spectra (Figure 1). For mDHFR^F134Z^ and mDHFR^V135Z^, the expected monoisotopic masses for the 2Nal-containing fragments were 685.3 *m*/*z* (residue 133–137) and 733.3 *m*/*z* (residues 133–137), respectively. The actual monoisotopic masses for the 2Nal-containing fragments were found to be 685.4 *m*/*z* and 733.4 *m*/*z* for mDHFR^F134Z^ and mDHFR^V135Z^, respectively (Figure 1). Masses corresponding to 2Nal-containing fragments were not observed in the mDHFR^WT^ spectra, indicating that the 2Nal-containing fragment for each variant is unique and each arises from *in vivo* incorporation of 2Nal at the correct position. Also, the corresponding wild-type fragment and fragments pertaining to misincorporation of Phe and Trp were not observed in the mutant spectra. These results indicate that 2Nal was successfully incorporated into target sites with high fidelity.

### 2.4. Computational Analysis and Circular Dichroism Spectroscopy of mDHFR Variants Containing 2Nal

Assuming that the hydrophobic core region of an enzyme is tightly packed, we hypothesized that incorporation of a bulky non-natural amino acid reduces the conformational stability of the core region due to the steric incompatibility. In order to test this hypothesis, computational analysis using RosettaDesign was performed to evaluate the steric compatibility of 2Nal at each incorporation site (Table 1). The RosettaDesign fa_rep output is the Lennard-Jones repulsive (LJR) score, which is a measure of the deviation of atom distances from optimality [67]. Higher scores reflect a scenario where atoms are closer than the distance preferred by van der Waals forces. The LJR score is a measure of the steric compatibility of a mutation and, for a fixed-backbone technique, also represents the available free energy to drive structural changes to a more stable protein conformation [68,69,70,71,72]. The RosettaDesign LJR score was previously used to evaluate the conformational stability of human copper, zinc superoxide dismutase and was used successfully to predict mutations for both favorable and unfavorable conformational interactions [73,74].

**Table 1:** Parameters derived from computational and crystal structure analysis.

Variant	WT	W113Z	V112Z	F134Z	V135Z	I51Z	V50Z
fa_rep ^1^ (kcal/mol)	14.1 ± 0.5	66.5 ± 0.7	121.3 ± 34.8	59.7 ± 2.3	77.9 ± 1.5	96.7 ± 0.6	121.4 ± 1.2
DTS (Å) ^2^	NA ^3^	9	12	6	7	8	8

^1^ Lennard-Jones repulsive score derived by computational analysis (RossettaDesign 3.4) using a fixed-backbone method using PDB ID: 2W3M. Standard errors are reported (*n* = 3); ^2^ Distance to the substrate. Distances represent the lowest edge-to-edge distance from the native residue to the substrate and are binned to a whole number; ^3^ Not applicable.

The degree of steric incompatibility (LJR score) is related to the size difference between the mutant and the native residue (Supplementary Information Figure S1 and Table 1). Mutants of adjacent sites (W113Z/V112Z, F134Z/V135Z and I51Z/V50Z) were compared pairwise to minimize effects associated with location-dependence [75,76]. It was observed that the mutation with the smaller SDMNR of each pair (W113Z, F134Z and I51Z) consistently exhibited a lower LJR score than its adjacent counterpart (V112Z, V135Z and V50Z) with the larger SDMNR (Table 1; Supplementary Information Figure S1). As would be expected, these results indicate that between mutants of adjacent sites, the mutation with the smaller SDMNR is more sterically compatible. In other words, the degree of steric incompatibility (LJR score) is related to the SDMNR. These results support our hypothesis that minimizing the SDMNR reduces steric incompatibility.

Far-UV circular dichroism spectroscopy was performed to qualitatively analyze the changes in secondary structure (Supplementary Information Figure S3). Due to the high β-sheet content, the mDHFR^WT^ spectrum is highlighted by a minimum at 212 nm, a β-sheet signature. The mDHFR^WT^ spectrum also exhibits a shoulder at 222 nm associated with α-helical structure. Regarding the mDHFR^W113Z^ and mDHFR^F134Z^ variants, a modest reduction in the signal at 212 nm and an increase signal at 222 nm were observed, indicating some change in secondary structure. Such structural changes might be attributed to steric instability upon 2Nal incorporation. CD spectra could not be obtained for mDHFR^V50Z^, mDHFR^I51Z^, mDHFR^V112Z^, and mDHFR^V135Z^ due to low expression and purification yields, suggesting these variants are structurally unstable and so are prone to degradation inside cells. We speculate that mDHFR^W113Z^ and mDHFR^F134Z^ variants are structurally more stable than other mDHFR variants (mDHFR^V50Z^, mDHFR^I51Z^, mDHFR^V112Z^, and mDHFR^V135Z^).

### 2.5. Evaluating the Effects of 2Nal Incorporation on mDHFR Substrate Binding

Next, we examined how the incorporation of 2Nal affected enzyme function. Substrate binding is the obligate step in all enzymatic reactions before biocatalysis can occur [77]. Since reduction of DHF by mDHFR follows Michaelis-Menten kinetics within the measured substrate concentration range (0–40 μM DHF), *K*_m_, the inverse measure of binding affinity, was used to evaluate the effect of 2Nal incorporation on the substrate-binding component of enzyme function. The *K*_m_ values for the conservative mutations W113Z and F134Z were statistically indistinguishable from that of mDHFR^WT^ (Student’s *t*-test; *p* > 0.05) (Table 2; Supplementary Information Figure S4). This indicates that the two conservative mutations did not significantly affect DHF binding affinity, though 2Nal incorporation caused the changes in secondary structure.

**Table 2:** Michaelis-Menten kinetic parameters for DHF reduction by mDHFR ^1^.

Variant	WT	W113Z	V112Z	F134Z	V135Z	I51Z	V50Z
*k*_cat_ (s^−1^)	3.66 ± 0.02	2.95 ± 0.002	0.029 ± 0.0001	1.46 ± 0.0002	0.32 ± 0.0004	0.028 ± 0.0002	0.020 ± 0.0002
*K*_m_ (μM)	1.94 ± 0.08	2.10 ± 0.19	2.61 ± 0.12	1.87 ± 0.19	1.47 ± 0.08	3.01 ± 0.11	3.43 ± 0.24
*k*_cat_/*K*_m_ (μM/s)	1.9 ± 0.1	1.4 ± 0.1	0.011 ± 0.001	0.78 ± 0.08	0.22 ± 0.01	0.0092 ± 0.001	0.0058 ± 0.0004
Rel. *k*_cat_/*K*_m_ (%) ^2^	100.0	74.5 ± 12.7	0.6 ± 0.05	41.4 ± 8.1	11.7 ± 1.2	0.5 ± 0.05	0.3 ± 0.04
|Δ*K*_m_| (μM) ^3^	NA	0.16	0.67	0.07	0.47	1.07	1.49

^1^ Reactions were conducted with DHF concentrations of 0–40 and 60 μM of NADPH. Standard errors are shown (*n* = 3); ^2^ Relative catalytic efficiency of the particular variant compared to that of mDHFR^WT^; ^3^ Absolute difference between the *K*_m_ of the mutant mDHFR and that of wild-type mDHFR.

The packed structure of the hydrophobic core is held together predominantly by van der Waals interactions, and strongly depends on both the steric compatibility and configuration of its residues [44,47,78]. Therefore, the hydrophobic core structure is highly sensitive to mutations that promote conformational strain or cavitations [42]. Moreover, enzyme-substrate binding affinity is sensitive to structural changes and can be affected by either a change to the binding site structure itself or by a change in the structure outside the binding site that alters the stability of the substrate-bound conformation [77,79,80]. DHF is known to interact with several residues inside the active site (I7, L22, E30, F31 and N64) via multiple non-covalent bonds [81,82]. As expected, a number of mutations have been shown to affect binding site structure and binding affinity of DHFR through the propagation of structural changes between the mutation and binding site [75,77,82,83,84,85,86,87]. Therefore, we suspect that there is a link between the steric compatibility of a mutation in the hydrophobic core and changes in substrate binding affinity.

To determine whether the steric compatibility of a mutation in the hydrophobic core is an important variable that affects substrate binding, variants of adjacent sites were compared pairwise. The LJR score did not trend with *K*_m_ itself. Instead, we observed that, between variants of adjacent sites, the LJR score trended with the absolute difference between the *K*_m_ of the variant and that of mDHFR^WT^ (|Δ*K*_m_|). 2Nal mutations with lower LJR scores (W113, F134 and I51) consistently resulted in a lower |Δ*K*_m_| than ones with higher LJR scores (V112, V135, V50) (Table 1 and Table 2). Also, the difference in the *K*_m_ values between variants of adjacent sites was found to be statistically significant (Student’s *t*-test; *p* < 0.05). Moreover, *K*_m_ and |Δ*K*_m_| did not trend or correlate with the distance of the mutation site from the active site (DTS) (Table 1 and Table 2), nor did they trend or correlate with ASA, which was kept relatively constant through a rigorous mutation selection process.

To determine whether there was a correlation between the LJR score and |Δ*K*_m_|, we calculated the Pearson’s correlation coefficient (PCC), *r*, which is used to evaluate the linearity between two variables. From this analysis, *r* was determined to be 0.831, and the corresponding *p*-value was found to be equal to 0.041. Since the *p*-value was less than 0.05 and the *r* value was greater than zero, the statistical analysis indicates that a statistically significant positive correlation is present between the LJR score and |Δ*K*_m_|. While the sample size was small, it was taken into account during calculation of the PCC and *p*-value [44,78,88]. Collectively, these results indicate that steric compatibility is predictive to the degree to which *K*_m_ changes. A similar phenomenon has been observed in a number of cases where the protein stability correlates either positively or negatively with ligand binding affinity [89]. Our results suggest that, for the variants, steric compatibility of the mutation plays a significant role in changing substrate-binding affinity.

### 2.6. Evaluating the Effect of 2Nal Incorporation on Catalytic Function

Next, we evaluated the effects of each mutation on catalytic function using two kinetic parameters: the substrate turnover rate (*k*_cat_) and catalytic efficiency (*k*_cat_/*K*_m_). While the conservative mutations, W113Z and F134Z, did not significantly change the substrate binding affinity (Table 2), they reduced the substrate turnover rate and the catalytic efficiency (Table 2; Supplementary Information Figure S5). Based on the inspection of the crystal structure of DHFR (PDB ID:2W3M), it is not apparent how these two mutations affected the catalytic efficiency. Both sites are located in the rigid β-sheet core of mDHFR and are not directly associated with loop regions that are essential for catalytic function [77]. However, since secondary structure changes were observed for these two mutations, we found out that preservation of the mDHFR core structure is important for catalytic function [90].

To evaluate the effects of the steric incompatibility caused by 2Nal incorporation on catalytic function, we compared the *k*_cat_ value of mDHFR variants of adjacent incorporation sites in a pairwise manner. Between the adjacent sites, mutations with the lower LJR scores consistently retained a higher substrate turnover rate and catalytic efficiency (Table 2). W113Z, F134Z and I51Z had 102-, 4.6- and 1.4-fold higher substrate turnover rates, respectively, than their counterpart variant containing Val to 2Nal mutation. The same trend was observed between the LJR score and catalytic efficiency (*k*_cat_/*K*_m_).

When we calculated the Pearson’s correlation coefficient for *k*_cat_ as a function of the LJR score, the *r* and corresponding *p*-value were determined to be −0.739 and 0.093, respectively. Since, *p* > 0.05, this indicates that the LJR score does not correlate linearly with *k*_cat_. Since the *r* value was relatively high (−1 ≤ *r* ≤ +1), we suspected that a monotonic correlation might be present instead. Therefore, we calculated the Spearman rho correlation coefficient, *ρ*, which was determined to be −0.886 with a corresponding *p*-value equal to 0.019. Judging from *ρ* and *p* values, *k*_cat_ has a decreasing monotonic trend with respect to the LJR score in a non-linear manner. A similar correlation was observed between *k*_cat_/*K*_m_ and the LJR score.

Steric incompatibility can affect enzyme function in a number of ways. Steric incompatibility can compromise enzyme function by disrupting global conformation resulting in severely misfolded or denatured enzymes. Steric incompatibility can also affect enzyme function by decreasing the dynamic motion of an enzyme. Protein dynamics have increasingly been shown to be important for enzyme function [91,92,93,94,95,96]. Structural features such as loop regions are known to mediate catalytic function of mDHFR. For example, the opening and occlusion of loop regions around the active site during the unbinding of product and NADP^+^ are rate-limiting steps in the catalytic pathway [39,77,94,95,97,98,99]. Mutation of G121 in DHFR has been shown to decrease the dynamic range of motion thus inhibiting catalysis [98,100,101]. Therefore, we suspect that the hydrophobic core mutations affect mDHFR function through one of these mechanisms. However, further studies involving structural analysis are necessary to clarify the appropriate mechanism.

## 3. Experimental Section

### 3.1. Materials

3-(2-naphthy)-alanine (2Nal) was obtained from Chem-Impex (Wood Dale, IL, USA). Nickel-nitrilotriaceticacid (Ni-NTA) affinity column resin and plasmid pQE16 were obtained from Qiagen (Valencia, CA, USA). Sequencing-grade trypsin was procured from Promega (Madison, WI, USA). ZipTip C18 was purchased from Millipore (Billerica, MA, USA). Dihydrofolate (DHF), nicotinamide adenine dinucleotide phosphate (NADPH), and isopropyl β-d-1-thiogalactopyranoside (IPTG) were obtained from Santa Cruz Biotechnologies (Dallas, TX, USA). All other chemicals, unless otherwise noted, were purchased from Sigma-Aldrich (St. Louis, MO, USA).

### 3.2. Expression and Purification of Wild-Type mDHFR and Its Variants

The plasmid pQE16-mDHFR^WT^-yPheRS^naph^ encoding wild-type DHFR (mDHFR^WT^) and engineered yeast phenylalanyl-tRNA/synthetase pair specific for 2Nal was transformed into the *E. coli* AFWK expression host, as reported previously [1]. For mDHFR^WT^ expression, the transformed expression host was cultured in M9-20AA media (6 g/L Na_2_HPO_4_, 3 g/L KH_2_PO_4_, 0.5 g/L NaCl, 0.1 mM CaCl_2_, 1 mM MgSO_4_, 0.4% glucose, 50 mg/L of each of the 20 natural amino acids) supplemented with 100 μg/mL ampicillin. The cells were grown to OD_600_ = 1.0 and induced with a final concentration of 1 mM IPTG. We used amber suppression technique to incorporate 2Nal into a specific site of mDHFR, as reported previously [1]. We performed a PCR-mutagenesis to introduce an amber codon to each of six sites (Val50, Ile51, Val112, Trp113, Phe134, and Val135) in the core region of mDHFR generating six plasmids (pQE16-XXAmb_mDHFR-yPheRS^naph^; XX denotes a residue mutated). With an aid of the engineered yeast phenylalanyl-tRNA/synthetase pair specific for 2Nal, 2Nal was site-specifically introduced to an amber codon site of each mDHFR variant. Cells were harvested after overnight induction at 32 °C. In order to express the mDHFR variants containing 2Nal, the *E. coli* AFWK host was co-transformed with the two plasmids pQE16-XXAmb_mDHFR-yPheRS^naph^ and pREP4-ytRNA^Phe^_CUA_UG_ as reported previously except mutation sites [1]. The cells were then grown in M9-20AA, supplemented with 100 μg/mL ampicillin and 50 μg/mL kanamycin, until OD_600_ = 1.0. Cells were gently washed twice by repeating centrifugation at 4500× *g* for 8 min and resuspended using 0.9% NaCl solution. After washing twice, the resulting cell pellet was resuspended in M9-17AA (6 g/L Na_2_HPO_4_, 3 g/L KH_2_PO_4_, 0.5 g/L NaCl, 0.1 mM CaCl_2_, 1 mM MgSO_4_, 0.4% glucose, 0.05 g/L of each of the natural amino acids except phenylalanine (25 μM), tryptophan (50 μM), and lysine (100 μM) as reported previously [24]. The M9-17AA was then supplemented with 3 mM of 2Nal. After incubation at 37 °C for 10 min, induction was initiated by the addition of IPTG (1 mM). The cells were induced overnight at 32 °C. Compared to wild-type mDHFR found in nature, the mDHFR used in this study has the extra residues MRGSGI at to the N-terminus and a hexahistidine purification tag at the C-terminus [1]. Purification of the mDHFR^WT^ and its variants was performed using Ni-NTA resin, according to manufacturer’s protocol (Qiagen, Valencia, CA, USA)—with the exception that 20 and 40 mM imidazole in the lysis and wash buffers, respectively [1]. The protein gel images were taken using a UVP BioSpectrum imager (Upland, CA, USA). Protein concentration was determined by absorbance measurements at 280 nm, using the calculated extinction coefficient of 24,750 cm^−1^·M^−1^ [1].

### 3.3. Confirmation of in Vivo 2Nal Incorporation by MALDI-TOF/MS

Site-specific incorporation of 2Nal into mDHFR was confirmed by MALDI-TOF/MS after digestion of each variant in elution buffer either by trypsin or chymotrypsin. The mDHFR^V50Z^, mDHFR^I51Z^, mDHFR^F134Z^, and mDHFR^V135Z^ variants were digested by trypsin. The mDHFR^V112Z^ and mDHFR^W113Z^ variants were digested by chymotrypsin [1]. After the enzymatic digestion at 37 °C overnight, the reaction was quenched by addition of trifluoroacetic acid. Digested peptides solutions were desalted using ZipTip C18 according to the manufacturer’s protocol. Twenty mg/mL of 2,5-dihydroxybenzoic acid and 2 mg/mL of l-fucose dissolved in 10% ethanol was used as the matrix for MS analysis using a Microflex MALDI-TOF/MS (Bruker, Billerica, MA, USA).

### 3.4. Dihydrofolate Reduction Kinetics

The eluted mDHFR was desalted on a PD-10 column (GE Healthcare, Pittsburgh, PA, USA) per manufacture’s protocol. The reaction kinetics of DHF conversion into tetrahydrofolate was monitored by measuring absorbance at 340 nm, using a Synergy four multimode microplate reader (BioTek, Winooski, VT, USA) according to manufacturer’s protocol (Sigma-Aldrich, St. Louis, MO, USA). To determine DHF reduction kinetic parameters, 60 μM of NADPH and varying concentrations of DHF (0–40 μM) were dissolved in MTEN buffer (50 mM 2-(*N*-morpholino)ethanesulfonic acid (MES), 25 mM tris(hydroxymethyl)aminomethane (Tris), 25 mM ethanolamine, and 800 mM sodium chloride, pH 7.5) supplemented with 0.1 mg/mL of bovine serum albumin. The mDHFR variant was added to a final concentration of 2 to 100 nM, depending on the variant. Bovine serum albumin (BSA) was added to each reaction at a final concentration of 0.1 mg/mL [102,103]. All measurements were performed in at least triplicates. Kinetic parameters *k*_cat_, *K*_m_, and *k*_cat_/*K*_m_ were obtained by fitting reaction rates with respect to substrate concentration against the Michaelis-Menten model [1,31,32,33,35,38,104]. The extinction coefficient for DHF conversion into tetrahydrofolate is 12,300 M^−1^/cm at 340 nm at 25 °C [102,103].

### 3.5. Circular Dichroism Spectroscopy

The purified mDHFR variants were diluted using 10 mM phosphate buffer to a final concentration of 2 μM and were subjected to circular dichroism spectroscopy using a Jasco J-710 spectropolarimeter. The reported spectrum for each sample was an average of at least five measurements, and the solvent background spectrum was subtracted.

### 3.6. Computational Analysis of Conformational Stability and Mutational Steric Compatibility

Computational analysis of the conformational stability of mDHFR upon NNAA incorporation was performed using the molecular modeling program RosettaDesign, version 3.4 [67,89]. The energy score output of RosettaDesign is a linear sum of the Lennard-Jones potential, the Lazaridis-Karplus solvation energy, an empirical hydrogen bonding potential, the internal energy of sidechain rotamers, the Coulombic electrostatic energy potential, the energetic favorability of a specific amino acid based on φ/ψ angles, and a unique reference energy for each amino acid [67,68,89]. For a given crystal structure, RosettaDesign uses a Monte Carlo method to simulate residue annealing by scanning through a large number of rotamers for a given non-natural amino acid (from the SwissSidechain database) to minimize the energy score output. This analysis used a fixed-backbone technique [105]. A fixed backbone technique was previously used successfully for computational design of proteins [73,74,106,107,108,109,110]. Since a crystal structure for wild-type mDHFR bound to either DHF or folate (FOL) is not available, the human DHFR (hDHFR) structure (PDB ID: 2W3M) was used instead. The hDHFR structure is highly homologous to that of mDHFR with a greater than 95% sequence homology [32,40].

## 4. Conclusions

In this study, we used 2Nal and mDHFR as a model case to investigate how the incorporation of a bulky, hydrophobic non-natural amino acid into the hydrophobic core of an enzyme affects the enzyme structure and catalytic properties. We hypothesized that mutations that are intended to minimize structural changes may be able to minimize effect on mDHFR enzyme function. As expected, the two conservative mutations W113Z and F134Z led to the modest reduction in catalytic efficiency, while non-conservative mutations V50Z, I51Z, V112Z, and V135Z reduced the catalytic efficiency into a level less than 12% of that of mDHFR^WT^. The steric incompatibility (LJR score calculated by the by RosettaDesign of a given 2Nal substitution was found to have a linear positive correlation with |Δ*K*_m_|, the absolute difference between the *K*_m_ of the variant and that of mDHFR^WT^. This correlation is likely due to the fact that substrate-binding affinity is closely related to the structure and conformation of an enzyme. Statistical analysis also revealed that *k*_cat_ and *k*_cat_/*K*_m_ have a negative non-linear monotonic correlation with the LJR score, suggesting that both the binding and catalytic component of mDHFR are dependent on the steric compatibility of the 2Nal mutation. Considering all the results obtained from this study, we conclude that the NNAA incorporation minimizing steric instability has minimal effects on enzyme catalytic properties. In other words, in order to minimize perturbation of the catalytic properties of an enzyme upon NNAA incorporation, researchers need to choose a mutation site minimizing steric instability. In order to further expand our research, we plan to investigate whether steric incompatibility is an important factor to estimate the degree of change in catalytic properties upon NNAA incorporation at non-hydrophobic core region of an enzyme, including enzyme surface, as a follow-up research.

## Supplementary Materials

Supplementary materials can be found at http://www.mdpi.com/1422-0067/16/09/22735/s1.
