# Supplementary material for: Effects of Non-Natural Amino Acid Incorporation into the Enzyme Core Region on Enzyme Structure and Function
Source: Int J Mol Sci. 2015 Sep 21;16(9):22735–53. doi: 10.3390/ijms160922735 (PMC4613333; doi:10.3390/ijms160922735)
Supplement: Supplementary file 1 [file ijms-16-22735-s001.pdf]

Supplementary Information

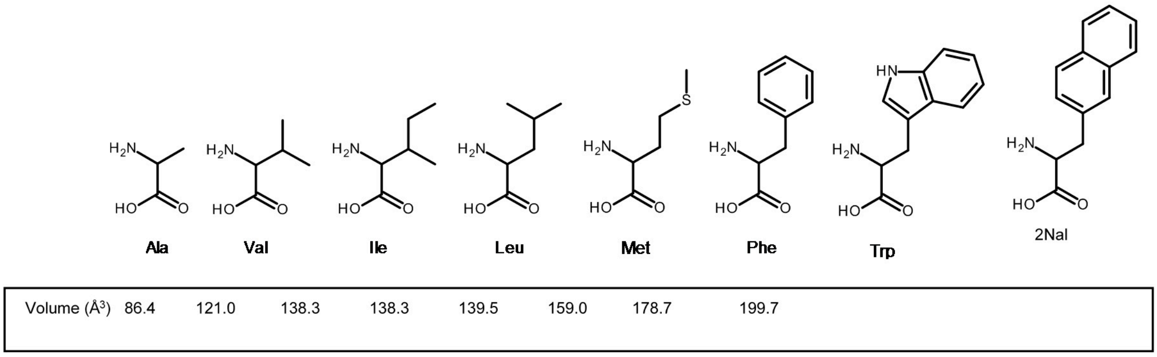

Figure S1. Structures and the calculated van der Waals volumes of hydrophobic amino acids in order of increasing size. Van der Waals volumes were calculated using the method reported previously [59].

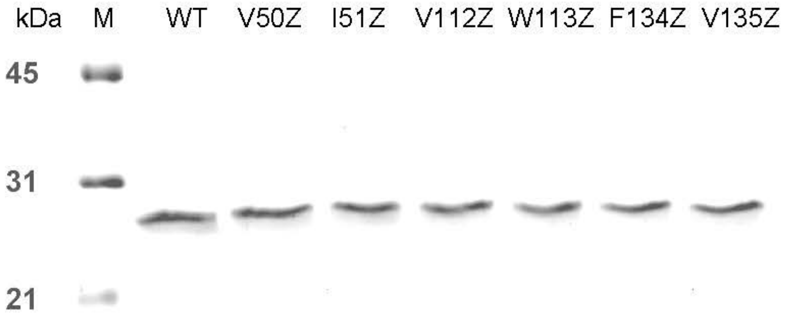

Figure S2. SDS-PAGE showing purified mDHFR^{WT} and hydrophobic core mutants. M, WT, and Z denote molecular weight markers, mDHFR^{WT} and 2Nal, respectively.

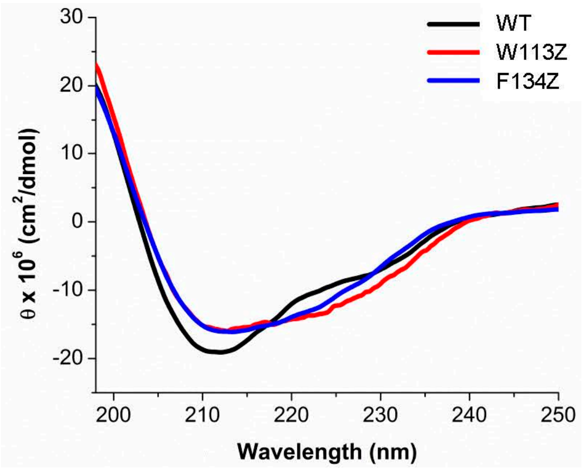

Figure S3. Evaluation of secondary structure changes by far-UV circular dichroism. Spectra for mDHFR^{WT} (WT; black), mDHFR^{W113Z} (W113Z; red), and mDHFR^{F134Z} (F134Z; blue) are shown over the wavelength range of 195–250 nm. Spectra were obtained by averaging at least $n = 5$ measurements.

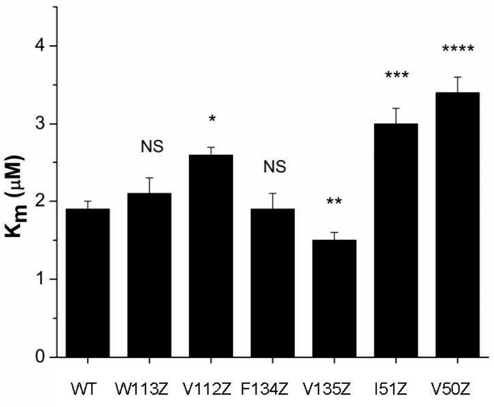

Figure S4. Michaelis-Menten constants (K_m) for DHF for $mDHFR^{WT}$ and the variants. K_m values derived from the Michaelis-Menten model, were based on saturation kinetics. WT denotes $mDHFR^{WT}$. Error bars denote the standard error ($n = 3$). Here Z denotes 2Nal. The Student's *t*-test (two-tailed and unpaired) *p*-values are for comparison of the variant with $mDHFR^{WT}$ (* $p = 0.001$; ** $p = 0.002$; *** $p = 0.0006$; and **** $p = 0.0005$). NS indicates that the difference is not significant.

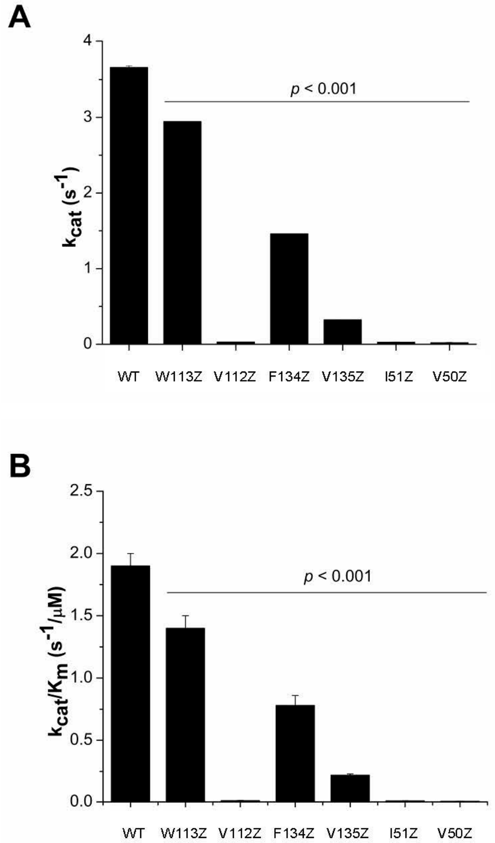

Figure S5. Substrate turnover rate (k_{cat}) and catalytic efficiency (k_{cat}/K_m) for DHF reduction for $mDHFR^{WT}$ and its variants. The kinetic parameters (**A**) k_{cat} and (**B**) k_{cat}/K_m were derived from the Michaelis-Menten equation. Values were obtained from averaging values from at least $n = 3$ samples. Error bars denote the standard error. Here Z denotes 2Nal. The Student's *t*-test (two-tailed and unpaired) was used to calculate *p*-values for comparison between variants with $mDHFR^{WT}$.
